# 15,15-Diphenyl-2,3,4,5,6,8,9,11,12-octa­hydro­imidazo[2,1-*h*][1,4,12]trioxa[7]thia­[9]azacyclo­tetra­decin-14(15*H*)-one

**DOI:** 10.1107/S2414314623001256

**Published:** 2023-02-17

**Authors:** Walid Guerrab, Abderrazzak El Moutaouakil Ala Allah, Abdulsalam Alsubari, Joel T Mague, Youssef Ramli

**Affiliations:** a Laboratory of Medicinal Chemistry, Drug Sciences Research Center, Faculty of Medicine and Pharmacy, Mohammed V University in Rabat, Morocco; b Laboratory of Medicinal Chemistry, Faculty of Clinical Pharmacy, 21 September University, Yemen; c Department of Chemistry, Tulane University, New Orleans, LA 70118, USA; University of Aberdeen, United Kingdom

**Keywords:** crystal structure, thio­hydantoin, crown ether

## Abstract

The title mol­ecule, C_23_H_26_N_2_O_4_S, adopts a cup-shaped conformation. In the crystal, layers lying parallel to the *ab* plane are formed by C—H⋯O hydrogen bonds and C—H⋯π(ring) inter­actions. The layers stack along the *c*-axis direction through normal van der Waals inter­actions.

## Abstract 2

The title mol­ecule adopts a cup-shaped conformation. In the crystal, layers lying parallel to the *ab* plane are formed by C—H⋯O hydrogen bonds and C—H⋯π(ring) inter­actions.

## Graphical abstract

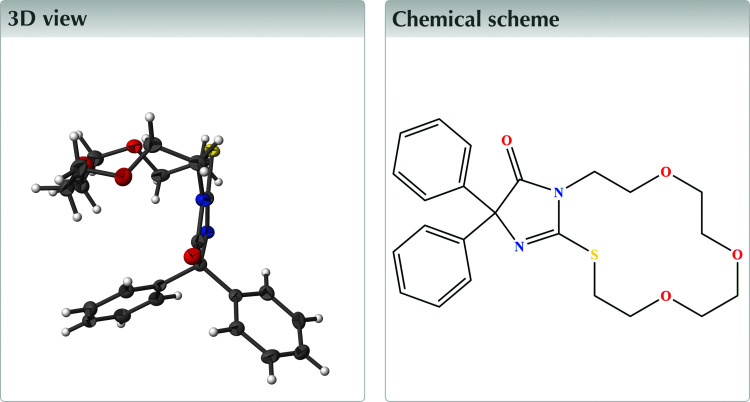

## Structure description

**Figure 1:**
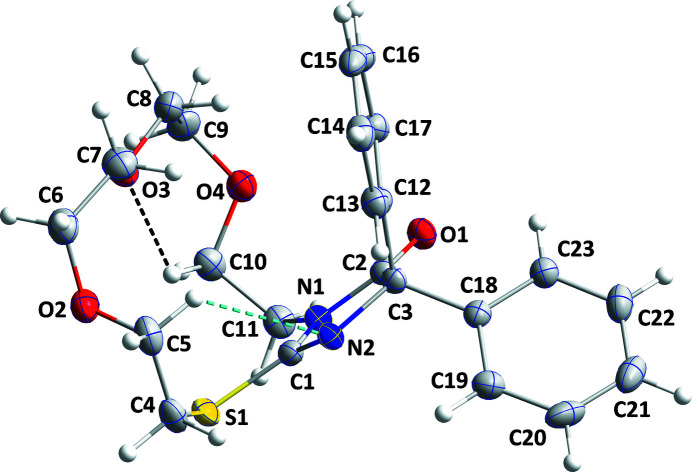
The title mol­ecule with 50% probability ellipsoids. Intra­molecular hydrogen bonds are depicted by dashed lines.

Compounds containing the thio­hydantoin scaffold exhibit many pharmacological activities, including anti­microbial, anti­carcinogenic, anti-inflammatory, anti­bacterial, anti-androgen and anti-diabetic effects (Meusel *et al.*, 2004 ▸; Tomasic *et al.*, 2009 ▸; Scholl *et al.* 1999 ▸; Vengurlekar *et al.* 2012 ▸; Jain *et al.* 2013 ▸; Efsta­thiou *et al.* 2015 ▸). As part of our ongoing work in this area (Guerrab *et al.* 2022*a* ▸,*b* ▸, 2023 ▸), the title compound (Fig. 1 ▸) was prepared and its crystal structure is reported here.

**Figure 2:**
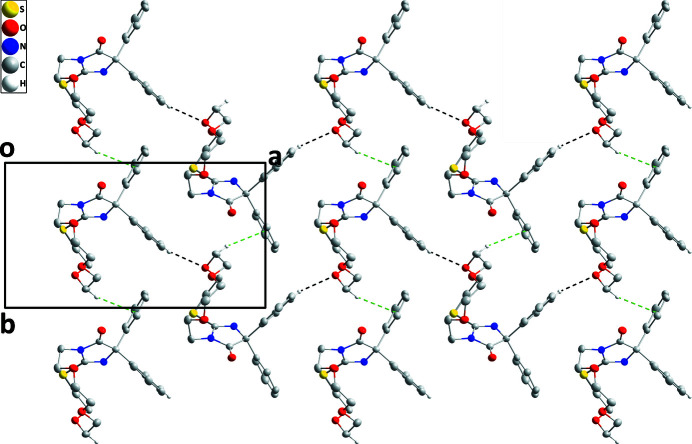
Plan view of the layer structure viewed along the *c*-axis direction. C—H⋯O hydrogen bonds and C—H⋯π(ring) inter­actions are shown by black and green dashed lines, respectively.

**Figure 3:**
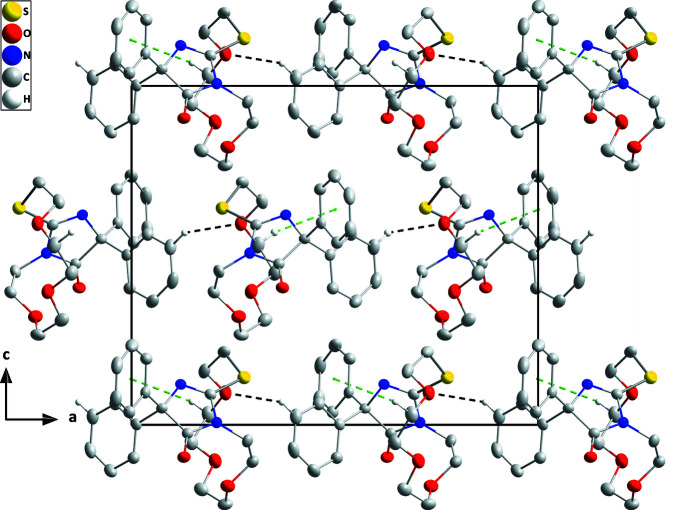
Elevation view of the layer structure seen along the *b*-axis direction with inter­molecular inter­actions depicted as in Fig. 2 ▸.

The mol­ecule adopts a cup-shaped conformation with the five-membered ring as the base and the C12–C17 benzene ring and the crown ether ring as the sides. This conformation is likely due to packing considerations since the three-dimensional structure is fairly compact with this arrangement (Fig. 2 ▸) but it may also be aided by the C5—H5*B*⋯N2 hydrogen bond (Table 1 ▸ and Fig. 1 ▸). The five-membered ring is almost planar (r.m.s. deviation = 0.009 Å) and the C12–C17 and C18–C23 benzene rings are inclined to it by 62.10 (7) and 61.35 (9)°, respectively. The conformation of the crown ether ring places S1, O2 and O3 pointing away from the center of the mol­ecule, but O4 points towards it. Intra­molecular C—H⋯O and C—H⋯N inter­actions occur (Table 1 ▸). In the crystal, zigzag chains of mol­ecules extending along the *a*-axis direction are linked by C14—H14⋯O2 hydrogen bonds and these are connected into layers lying parallel to the *ab* plane by C6—H6*A*⋯*Cg*3 inter­actions (Table 1 ▸ and Fig. 2 ▸). The layers stack along the *c*-axis direction with normal van der Waals contacts (Fig. 3 ▸).

## Synthesis and crystallization

To a solution of 5,5-diphenyl-2-thioxoimidazolidin-4-one (500 mg, 1.86 mmol), one equivalent of 1-chloro-2-{2-[2-(2-chloro­eth­oxy)eth­oxy]eth­oxy}ethane (365 µl, 1.86 mmol) dissolved in absolute di­methyl­formamide (DMF, 10 ml) was added and the resulting solution heated under reflux for 4 h in the presence of two equivalents of K_2_CO_3_ (513 mg, 3.72 mmol). The reaction mixture was filtered while hot, and the solvent evaporated under reduced pressure. The residue obtained was dried and recrystallized from ethanol solution to yield colourless blocks of the title compound (Guerrab *et al.*, 2018 ▸).

## Refinement

Crystal data, data collection and structure refinement details are summarized in Table 2 ▸. The crystal studied was refined as a two-component inversion twin with a refined BASF value of 0.25 (7).

## Supplementary Material

Crystal structure: contains datablock(s) global, I. DOI: 10.1107/S2414314623001256/hb4423sup1.cif

Structure factors: contains datablock(s) I. DOI: 10.1107/S2414314623001256/hb4423Isup2.hkl

CCDC reference: 2241205

Additional supporting information:  crystallographic information; 3D view; checkCIF report

## Figures and Tables

**Table 1:** Hydrogen-bond geometry (Å, °) *Cg*3 is the centroid of the C18–C23 benzene ring.

*D*—H⋯*A*	*D*—H	H⋯*A*	*D*⋯*A*	*D*—H⋯*A*
C5—H5*B*⋯N2	0.99	2.59	3.233 (3)	123
C10—H10*B*⋯O3	0.99	2.50	3.162 (3)	124
C14—H14⋯O2^i^	0.95	2.56	3.397 (3)	148
C6—H6*A*⋯*Cg*3^ii^	0.99	2.84	3.792 (3)	162

Symmetry codes: (i) 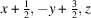 ; (ii) 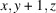 .

**Table 2:** Experimental details

Crystal data	
Chemical formula	C_23_H_26_N_2_O_4_S
*M*_r_	426.52
Crystal system, space group	Orthorhombic, *P* *n* *a*2_1_
Temperature (K)	150
*a*, *b*, *c* (Å)	16.6007 (17), 9.2362 (10), 13.8439 (14)
*V* (Å^3^)	2122.6 (4)
*Z*	4
Radiation type	Mo *K*α
μ (mm^−1^)	0.19
Crystal size (mm)	0.28 × 0.24 × 0.17

Data collection	
Diffractometer	Bruker SMART APEX CCD
Absorption correction	Multi-scan (*SADABS*; Krause *et al.*, 2015 ▸)
*T*_min_, *T*_max_	0.84, 0.97
No. of measured, independent and observed [*I* > 2σ(*I*)] reflections	19152, 5219, 4568
*R*_int_	0.033
(sin θ/λ)_max_ (Å^−1^)	0.667

Refinement	
*R*[*F* ^2^ > 2σ(*F* ^2^)], *wR*(*F* ^2^), *S*	0.036, 0.083, 1.03
No. of reflections	5219
No. of parameters	272
No. of restraints	1
H-atom treatment	H-atom parameters constrained
Δρ_max_, Δρ_min_ (e Å^−3^)	0.42, −0.15
Absolute structure	Refined as an inversion twin
Absolute structure parameter	0.25 (7)

Computer programs: *APEX3* (Bruker, 2016 ▸), *SAINT* (Bruker, 2016 ▸), *SHELXT* (Sheldrick, 2015*a* ▸), *SHELXL2018/1* (Sheldrick, 2015*b* ▸), *DIAMOND* (Brandenburg & Putz, 2012 ▸), *SHELXTL* (Sheldrick, 2008 ▸).

## full crystallographic data

### Crystal data

**Table:**

C_23_H_26_N_2_O_4_S	*D*_x_ = 1.335 Mg m^−^^3^
*M**_r_* = 426.52	Mo *K*α radiation, λ = 0.71073 Å
Orthorhombic, *P**n**a*2_1_	Cell parameters from 8169 reflections
*a* = 16.6007 (17) Å	θ = 2.5–28.1°
*b* = 9.2362 (10) Å	µ = 0.19 mm^−^^1^
*c* = 13.8439 (14) Å	*T* = 150 K
*V* = 2122.6 (4) Å^3^	Block, colourless
*Z* = 4	0.28 × 0.24 × 0.17 mm
*F*(000) = 904	

### Data collection

**Table:**

Bruker Smart APEX CCD diffractometer	5219 independent reflections
Radiation source: fine-focus sealed tube	4568 reflections with *I* > 2σ(*I*)
Graphite monochromator	*R*_int_ = 0.033
Detector resolution: 8.3333 pixels mm^-1^	θ_max_ = 28.3°, θ_min_ = 2.5°
ω scans	*h* = −21→22
Absorption correction: multi-scan (*SADABS*; Krause *et al.*, 2015)	*k* = −12→11
*T*_min_ = 0.84, *T*_max_ = 0.97	*l* = −18→18
19152 measured reflections	

### Refinement

**Table:**

Refinement on *F*^2^	Secondary atom site location: difference Fourier map
Least-squares matrix: full	Hydrogen site location: inferred from neighbouring sites
*R*[*F*^2^ > 2σ(*F*^2^)] = 0.036	H-atom parameters constrained
*wR*(*F*^2^) = 0.083	*w* = 1/[σ^2^(*F*_o_^2^) + (0.0489*P*)^2^] where *P* = (*F*_o_^2^ + 2*F*_c_^2^)/3
*S* = 1.03	(Δ/σ)_max_ < 0.001
5219 reflections	Δρ_max_ = 0.42 e Å^−^^3^
272 parameters	Δρ_min_ = −0.15 e Å^−^^3^
1 restraint	Absolute structure: Refined as an inversion twin.
Primary atom site location: dual	Absolute structure parameter: 0.25 (7)

### Special details

**Table:**

Experimental. The diffraction data were collected in three sets of 363 frames (0.5° width in ω) at φ = 0, 120 and 240°. A scan time of 30 sec/frame was used.
Geometry. All esds (except the esd in the dihedral angle between two l.s. planes) are estimated using the full covariance matrix. The cell esds are taken into account individually in the estimation of esds in distances, angles and torsion angles; correlations between esds in cell parameters are only used when they are defined by crystal symmetry. An approximate (isotropic) treatment of cell esds is used for estimating esds involving l.s. planes.
Refinement. Refinement of F^2^ against ALL reflections. The weighted R-factor wR and goodness of fit S are based on F^2^, conventional R-factors R are based on F, with F set to zero for negative F^2^. The threshold expression of F^2^ > 2sigma(F^2^) is used only for calculating R-factors(gt) etc. and is not relevant to the choice of reflections for refinement. R-factors based on F^2^ are statistically about twice as large as those based on F, and R- factors based on ALL data will be even larger. H-atoms attached to carbon were placed in calculated positions (C—H = 0.95 - 0.99 Å). All were included as riding contributions with isotropic displacement parameters 1.2 - 1.5 times those of the attached atoms. Refinement in *Pna*2_1_ with *SHELXL* resulted in a Flack parameter of *ca* 0.25 and the message `inversion twin or centrosymmetric space group'. As the intensity statistics and *PLATON* ADSYMM did not support a centrosymmetric space group, the refinement was finished in *Pna*2_1_ treating the model as an inversion twin with the addition of the instructions TWIN and BASF 0.25. The refined value of BASF was 0.25 (7).

### Fractional atomic coordinates and isotropic or equivalent isotropic displacement parameters (Å^2^)

**Table:**

	*x*	*y*	*z*	*U*_iso_*/*U*_eq_	
S1	0.22326 (3)	0.46089 (6)	0.63964 (4)	0.02584 (14)	
O1	0.37107 (10)	0.15624 (16)	0.40483 (11)	0.0262 (3)	
O2	0.26905 (9)	0.77584 (18)	0.59446 (13)	0.0305 (4)	
O3	0.29439 (10)	0.71630 (18)	0.39376 (13)	0.0332 (4)	
O4	0.26344 (11)	0.41717 (17)	0.31948 (13)	0.0311 (4)	
N1	0.29066 (11)	0.2903 (2)	0.50682 (14)	0.0221 (4)	
N2	0.37902 (11)	0.37283 (19)	0.61953 (13)	0.0220 (4)	
C1	0.30600 (13)	0.3719 (2)	0.58992 (16)	0.0212 (4)	
C2	0.36224 (13)	0.2330 (2)	0.47512 (15)	0.0209 (4)	
C3	0.42545 (13)	0.2866 (2)	0.54857 (15)	0.0201 (4)	
C4	0.27256 (15)	0.5931 (3)	0.71517 (17)	0.0293 (5)	
H4A	0.231310	0.644885	0.753541	0.035*	
H4B	0.308662	0.542256	0.760888	0.035*	
C5	0.32084 (14)	0.7016 (3)	0.65901 (19)	0.0310 (6)	
H5A	0.346442	0.771405	0.703764	0.037*	
H5B	0.363887	0.651719	0.622371	0.037*	
C6	0.31097 (16)	0.8699 (3)	0.5306 (2)	0.0346 (6)	
H6A	0.351600	0.924720	0.568061	0.041*	
H6B	0.272262	0.940579	0.503540	0.041*	
C7	0.35286 (15)	0.7933 (3)	0.4482 (2)	0.0350 (6)	
H7A	0.380465	0.864852	0.406480	0.042*	
H7B	0.393736	0.725386	0.473819	0.042*	
C8	0.32854 (16)	0.6518 (3)	0.30959 (19)	0.0326 (6)	
H8A	0.378561	0.599576	0.327050	0.039*	
H8B	0.342461	0.727969	0.262161	0.039*	
C9	0.26965 (16)	0.5486 (3)	0.26582 (19)	0.0341 (6)	
H9A	0.216034	0.595253	0.262729	0.041*	
H9B	0.286596	0.525951	0.198937	0.041*	
C10	0.20222 (15)	0.4167 (3)	0.39115 (18)	0.0302 (5)	
H10A	0.148380	0.414019	0.360436	0.036*	
H10B	0.205884	0.505135	0.431330	0.036*	
C11	0.21474 (14)	0.2835 (3)	0.45276 (18)	0.0268 (5)	
H11A	0.169351	0.273990	0.498704	0.032*	
H11B	0.215005	0.196608	0.410870	0.032*	
C12	0.48827 (13)	0.3867 (2)	0.50146 (16)	0.0205 (4)	
C13	0.53956 (14)	0.4642 (2)	0.56242 (18)	0.0263 (5)	
H13	0.535967	0.451446	0.630374	0.032*	
C14	0.59561 (15)	0.5596 (2)	0.52449 (19)	0.0312 (6)	
H14	0.630297	0.611768	0.566506	0.037*	
C15	0.60130 (15)	0.5791 (3)	0.42553 (19)	0.0330 (6)	
H15	0.639361	0.645390	0.399664	0.040*	
C16	0.55158 (16)	0.5020 (3)	0.36486 (19)	0.0330 (6)	
H16	0.555677	0.514677	0.296938	0.040*	
C17	0.49500 (14)	0.4052 (3)	0.40275 (17)	0.0272 (5)	
H17	0.461081	0.351902	0.360427	0.033*	
C18	0.46593 (13)	0.1555 (2)	0.59558 (16)	0.0218 (5)	
C19	0.46380 (15)	0.1332 (3)	0.69500 (18)	0.0285 (5)	
H19	0.438323	0.201865	0.735956	0.034*	
C20	0.49917 (17)	0.0100 (3)	0.7340 (2)	0.0377 (7)	
H20	0.498233	−0.004434	0.801960	0.045*	
C21	0.53572 (16)	−0.0920 (3)	0.6754 (2)	0.0374 (6)	
H21	0.558427	−0.177220	0.702531	0.045*	
C22	0.53873 (15)	−0.0684 (3)	0.5777 (2)	0.0334 (6)	
H22	0.563913	−0.137691	0.536929	0.040*	
C23	0.50554 (15)	0.0549 (3)	0.53801 (18)	0.0262 (5)	
H23	0.509812	0.071219	0.470452	0.031*	

### Atomic displacement parameters (Å^2^)

**Table:**

	*U*^11^	*U*^22^	*U*^33^	*U*^12^	*U*^13^	*U*^23^
S1	0.0209 (2)	0.0282 (3)	0.0284 (3)	0.0015 (2)	0.0053 (2)	−0.0022 (3)
O1	0.0308 (9)	0.0251 (8)	0.0227 (8)	0.0017 (7)	−0.0012 (7)	−0.0063 (7)
O2	0.0252 (8)	0.0259 (9)	0.0403 (10)	0.0009 (7)	0.0014 (7)	−0.0011 (7)
O3	0.0290 (9)	0.0309 (9)	0.0398 (10)	−0.0008 (7)	0.0031 (8)	−0.0067 (8)
O4	0.0376 (10)	0.0279 (9)	0.0279 (9)	0.0030 (7)	0.0015 (8)	−0.0024 (7)
N1	0.0206 (9)	0.0232 (9)	0.0224 (9)	−0.0002 (7)	−0.0003 (7)	−0.0022 (8)
N2	0.0220 (9)	0.0237 (9)	0.0203 (10)	0.0015 (7)	0.0013 (7)	−0.0029 (7)
C1	0.0239 (11)	0.0186 (10)	0.0212 (11)	0.0000 (8)	0.0025 (9)	0.0006 (9)
C2	0.0230 (11)	0.0189 (10)	0.0207 (10)	−0.0004 (8)	−0.0002 (9)	0.0007 (8)
C3	0.0229 (11)	0.0191 (10)	0.0183 (10)	0.0010 (8)	−0.0003 (8)	−0.0027 (8)
C4	0.0288 (13)	0.0347 (13)	0.0243 (12)	0.0083 (10)	−0.0011 (10)	−0.0085 (10)
C5	0.0243 (12)	0.0293 (12)	0.0394 (15)	0.0043 (9)	−0.0049 (10)	−0.0108 (10)
C6	0.0313 (13)	0.0232 (12)	0.0493 (17)	−0.0033 (10)	0.0038 (12)	−0.0042 (11)
C7	0.0315 (13)	0.0313 (13)	0.0420 (15)	−0.0067 (11)	0.0035 (11)	−0.0012 (12)
C8	0.0366 (14)	0.0284 (13)	0.0328 (14)	0.0058 (11)	0.0051 (11)	0.0039 (11)
C9	0.0403 (15)	0.0377 (15)	0.0244 (13)	0.0057 (11)	−0.0031 (11)	0.0031 (10)
C10	0.0265 (12)	0.0375 (14)	0.0267 (12)	0.0032 (10)	−0.0035 (10)	−0.0027 (11)
C11	0.0214 (11)	0.0293 (12)	0.0298 (12)	−0.0037 (9)	−0.0046 (9)	−0.0051 (10)
C12	0.0202 (10)	0.0161 (10)	0.0251 (11)	0.0036 (8)	0.0018 (9)	−0.0001 (8)
C13	0.0254 (12)	0.0234 (11)	0.0301 (12)	0.0002 (9)	−0.0004 (10)	−0.0049 (10)
C14	0.0252 (12)	0.0228 (11)	0.0455 (15)	−0.0013 (9)	0.0007 (11)	−0.0050 (11)
C15	0.0233 (12)	0.0261 (12)	0.0495 (17)	0.0021 (10)	0.0071 (11)	0.0114 (11)
C16	0.0298 (13)	0.0373 (14)	0.0318 (13)	0.0056 (11)	0.0056 (10)	0.0116 (11)
C17	0.0253 (12)	0.0302 (13)	0.0262 (12)	0.0021 (9)	−0.0005 (10)	0.0024 (10)
C18	0.0182 (10)	0.0214 (10)	0.0259 (11)	−0.0023 (8)	−0.0016 (9)	0.0008 (9)
C19	0.0262 (13)	0.0341 (13)	0.0253 (12)	−0.0017 (10)	0.0012 (9)	0.0007 (10)
C20	0.0306 (14)	0.0514 (16)	0.0312 (14)	−0.0049 (12)	−0.0017 (11)	0.0191 (13)
C21	0.0243 (13)	0.0309 (13)	0.0569 (17)	0.0005 (11)	−0.0038 (11)	0.0162 (12)
C22	0.0246 (12)	0.0258 (12)	0.0497 (17)	0.0046 (10)	−0.0031 (12)	0.0004 (12)
C23	0.0252 (12)	0.0259 (12)	0.0274 (12)	0.0015 (9)	0.0003 (9)	−0.0007 (10)

### Geometric parameters (Å, º)

**Table:**

S1—C1	1.742 (2)	C9—H9A	0.9900
S1—C4	1.804 (2)	C9—H9B	0.9900
O1—C2	1.213 (3)	C10—C11	1.512 (3)
O2—C5	1.417 (3)	C10—H10A	0.9900
O2—C6	1.421 (3)	C10—H10B	0.9900
O3—C7	1.420 (3)	C11—H11A	0.9900
O3—C8	1.426 (3)	C11—H11B	0.9900
O4—C10	1.420 (3)	C12—C17	1.382 (3)
O4—C9	1.427 (3)	C12—C13	1.396 (3)
N1—C2	1.373 (3)	C13—C14	1.385 (3)
N1—C1	1.399 (3)	C13—H13	0.9500
N1—C11	1.467 (3)	C14—C15	1.385 (4)
N2—C1	1.280 (3)	C14—H14	0.9500
N2—C3	1.481 (3)	C15—C16	1.376 (4)
C2—C3	1.543 (3)	C15—H15	0.9500
C3—C18	1.531 (3)	C16—C17	1.399 (4)
C3—C12	1.538 (3)	C16—H16	0.9500
C4—C5	1.501 (4)	C17—H17	0.9500
C4—H4A	0.9900	C18—C23	1.390 (3)
C4—H4B	0.9900	C18—C19	1.392 (3)
C5—H5A	0.9900	C19—C20	1.390 (4)
C5—H5B	0.9900	C19—H19	0.9500
C6—C7	1.512 (4)	C20—C21	1.383 (4)
C6—H6A	0.9900	C20—H20	0.9500
C6—H6B	0.9900	C21—C22	1.371 (4)
C7—H7A	0.9900	C21—H21	0.9500
C7—H7B	0.9900	C22—C23	1.379 (3)
C8—C9	1.494 (4)	C22—H22	0.9500
C8—H8A	0.9900	C23—H23	0.9500
C8—H8B	0.9900		
			
C1—S1—C4	100.98 (11)	C8—C9—H9A	109.2
C5—O2—C6	113.00 (18)	O4—C9—H9B	109.2
C7—O3—C8	111.80 (19)	C8—C9—H9B	109.2
C10—O4—C9	114.71 (19)	H9A—C9—H9B	107.9
C2—N1—C1	108.25 (18)	O4—C10—C11	107.34 (19)
C2—N1—C11	124.32 (19)	O4—C10—H10A	110.2
C1—N1—C11	126.81 (19)	C11—C10—H10A	110.2
C1—N2—C3	106.09 (18)	O4—C10—H10B	110.2
N2—C1—N1	116.06 (19)	C11—C10—H10B	110.2
N2—C1—S1	128.12 (17)	H10A—C10—H10B	108.5
N1—C1—S1	115.82 (16)	N1—C11—C10	111.8 (2)
O1—C2—N1	125.9 (2)	N1—C11—H11A	109.3
O1—C2—C3	129.4 (2)	C10—C11—H11A	109.3
N1—C2—C3	104.72 (17)	N1—C11—H11B	109.3
N2—C3—C18	111.83 (18)	C10—C11—H11B	109.3
N2—C3—C12	108.13 (16)	H11A—C11—H11B	107.9
C18—C3—C12	110.98 (17)	C17—C12—C13	119.0 (2)
N2—C3—C2	104.83 (16)	C17—C12—C3	123.3 (2)
C18—C3—C2	108.94 (17)	C13—C12—C3	117.72 (19)
C12—C3—C2	112.00 (17)	C14—C13—C12	120.4 (2)
C5—C4—S1	113.21 (17)	C14—C13—H13	119.8
C5—C4—H4A	108.9	C12—C13—H13	119.8
S1—C4—H4A	108.9	C13—C14—C15	120.3 (2)
C5—C4—H4B	108.9	C13—C14—H14	119.9
S1—C4—H4B	108.9	C15—C14—H14	119.9
H4A—C4—H4B	107.7	C16—C15—C14	119.7 (2)
O2—C5—C4	109.02 (19)	C16—C15—H15	120.1
O2—C5—H5A	109.9	C14—C15—H15	120.1
C4—C5—H5A	109.9	C15—C16—C17	120.3 (2)
O2—C5—H5B	109.9	C15—C16—H16	119.8
C4—C5—H5B	109.9	C17—C16—H16	119.8
H5A—C5—H5B	108.3	C12—C17—C16	120.3 (2)
O2—C6—C7	114.1 (2)	C12—C17—H17	119.9
O2—C6—H6A	108.7	C16—C17—H17	119.9
C7—C6—H6A	108.7	C23—C18—C19	118.7 (2)
O2—C6—H6B	108.7	C23—C18—C3	119.5 (2)
C7—C6—H6B	108.7	C19—C18—C3	121.7 (2)
H6A—C6—H6B	107.6	C20—C19—C18	119.6 (2)
O3—C7—C6	108.7 (2)	C20—C19—H19	120.2
O3—C7—H7A	109.9	C18—C19—H19	120.2
C6—C7—H7A	109.9	C21—C20—C19	121.0 (2)
O3—C7—H7B	109.9	C21—C20—H20	119.5
C6—C7—H7B	109.9	C19—C20—H20	119.5
H7A—C7—H7B	108.3	C22—C21—C20	119.1 (3)
O3—C8—C9	109.7 (2)	C22—C21—H21	120.4
O3—C8—H8A	109.7	C20—C21—H21	120.4
C9—C8—H8A	109.7	C21—C22—C23	120.6 (3)
O3—C8—H8B	109.7	C21—C22—H22	119.7
C9—C8—H8B	109.7	C23—C22—H22	119.7
H8A—C8—H8B	108.2	C22—C23—C18	120.8 (2)
O4—C9—C8	112.3 (2)	C22—C23—H23	119.6
O4—C9—H9A	109.2	C18—C23—H23	119.6
			
C3—N2—C1—N1	2.5 (2)	C2—N1—C11—C10	−93.2 (3)
C3—N2—C1—S1	−176.97 (17)	C1—N1—C11—C10	76.6 (3)
C2—N1—C1—N2	−2.0 (3)	O4—C10—C11—N1	65.4 (2)
C11—N1—C1—N2	−173.2 (2)	N2—C3—C12—C17	−125.5 (2)
C2—N1—C1—S1	177.53 (15)	C18—C3—C12—C17	111.5 (2)
C11—N1—C1—S1	6.3 (3)	C2—C3—C12—C17	−10.5 (3)
C4—S1—C1—N2	17.3 (2)	N2—C3—C12—C13	53.2 (2)
C4—S1—C1—N1	−162.16 (17)	C18—C3—C12—C13	−69.8 (2)
C1—N1—C2—O1	179.9 (2)	C2—C3—C12—C13	168.20 (18)
C11—N1—C2—O1	−8.7 (4)	C17—C12—C13—C14	0.8 (3)
C1—N1—C2—C3	0.5 (2)	C3—C12—C13—C14	−177.91 (19)
C11—N1—C2—C3	172.0 (2)	C12—C13—C14—C15	0.1 (3)
C1—N2—C3—C18	−119.9 (2)	C13—C14—C15—C16	−0.7 (4)
C1—N2—C3—C12	117.66 (19)	C14—C15—C16—C17	0.5 (4)
C1—N2—C3—C2	−2.0 (2)	C13—C12—C17—C16	−1.0 (3)
O1—C2—C3—N2	−178.5 (2)	C3—C12—C17—C16	177.6 (2)
N1—C2—C3—N2	0.8 (2)	C15—C16—C17—C12	0.4 (4)
O1—C2—C3—C18	−58.6 (3)	N2—C3—C18—C23	173.82 (19)
N1—C2—C3—C18	120.68 (19)	C12—C3—C18—C23	−65.3 (3)
O1—C2—C3—C12	64.5 (3)	C2—C3—C18—C23	58.4 (3)
N1—C2—C3—C12	−116.16 (19)	N2—C3—C18—C19	−5.8 (3)
C1—S1—C4—C5	64.68 (19)	C12—C3—C18—C19	115.1 (2)
C6—O2—C5—C4	−173.52 (18)	C2—C3—C18—C19	−121.2 (2)
S1—C4—C5—O2	59.4 (2)	C23—C18—C19—C20	−1.7 (4)
C5—O2—C6—C7	77.0 (3)	C3—C18—C19—C20	177.9 (2)
C8—O3—C7—C6	174.8 (2)	C18—C19—C20—C21	−0.8 (4)
O2—C6—C7—O3	59.3 (3)	C19—C20—C21—C22	1.8 (4)
C7—O3—C8—C9	168.7 (2)	C20—C21—C22—C23	−0.3 (4)
C10—O4—C9—C8	91.9 (3)	C21—C22—C23—C18	−2.2 (4)
O3—C8—C9—O4	−74.9 (3)	C19—C18—C23—C22	3.2 (4)
C9—O4—C10—C11	−169.19 (19)	C3—C18—C23—C22	−176.4 (2)

### Hydrogen-bond geometry (Å, º)

*Cg*3 is the centroid of the C18–C23 benzene ring.

**Table:**

*D*—H···*A*	*D*—H	H···*A*	*D*···*A*	*D*—H···*A*
C5—H5*B*···N2	0.99	2.59	3.233 (3)	123
C10—H10*B*···O3	0.99	2.50	3.162 (3)	124
C14—H14···O2^i^	0.95	2.56	3.397 (3)	148
C6—H6*A*···*Cg*3^ii^	0.99	2.84	3.792 (3)	162

Symmetry codes: (i) *x*+1/2, −*y*+3/2, *z*; (ii) *x*, *y*+1, *z*.
